# Prostate rhabdomyosarcoma in a young adult: Case report and literature review

**DOI:** 10.1016/j.eucr.2021.101745

**Published:** 2021-06-14

**Authors:** Ousmane Sow, Alioune Sarr, Dibor Niang, Aboubacry Mbow, Yaya Sow, Alain Khassim Ndoye

**Affiliations:** a Urology Andrology Department, Aristide Le Dantec Hospital, Dakar, Senegal; b Department of Pathologists, Gaston Berger University, Saint-Louis, Senegal

**Keywords:** Adult, Rhabdomyosarcoma, Chemotherapy, Radiotherapy

## Abstract

Primary rhabdomyosarcoma of the adult prostate is rare and associated with an aggressive clinical course. Combined modality therapy has resulted in marked improvement in survival rates and reduced surgical morbidity for children with these tumors. However, in adults the prognosis remains poor.We report on a case of prostate rhabdomyosarcoma in an adult approached with combined-modality treatment, with the administration of 9 courses of doxorubicin, vincristine and endoxan, and, subsequent radiotherapy to the prostaticbed. The patient remained free of progression of disease for about 1 year.

## Introduction

Prostate rhabdomyosarcoma (PR) is a rare mesenchymatous tumor and accounts for less than 1% of malignant prostate tumors.^1^ PR is rare in adults and has highly malignant potential.^1^ Several therapeutic approaches are used in the management of PR, such as radical surgery, radiotherapy and chemotherapy.We report the case of PR in a young adult treated with concurrent radio-chemotherapy and having given complete remission.

## Case presentation

**Fig. 1:**
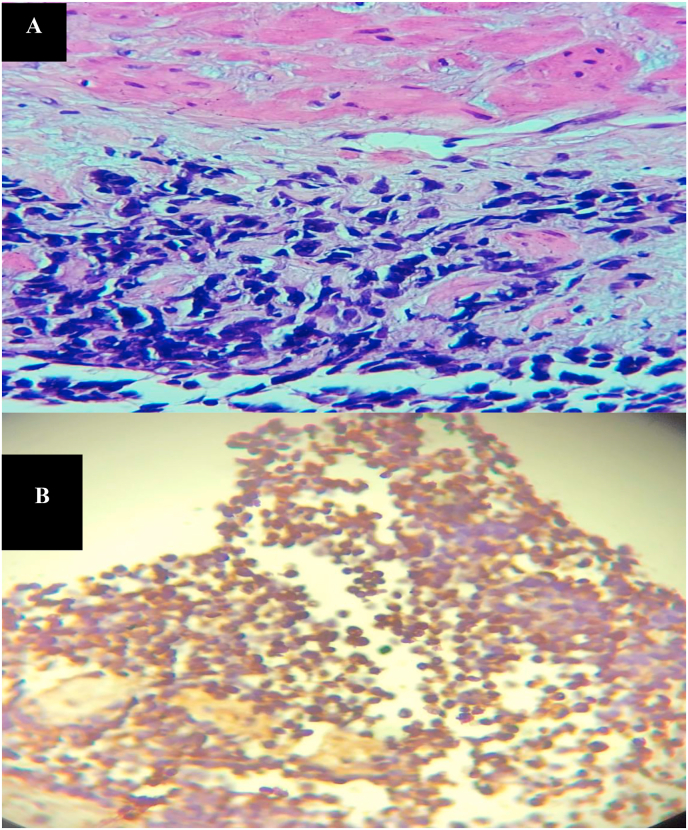
**(A)** Histology showing sheets of multinucleated cells in addition to small round cells (Haematoxylin-Eosin staining x 400) **(B)** The round cells demonstrated immunohistochemical positivity for myogenin (myogenin immunostaining x 100).

**Fig. 2:**
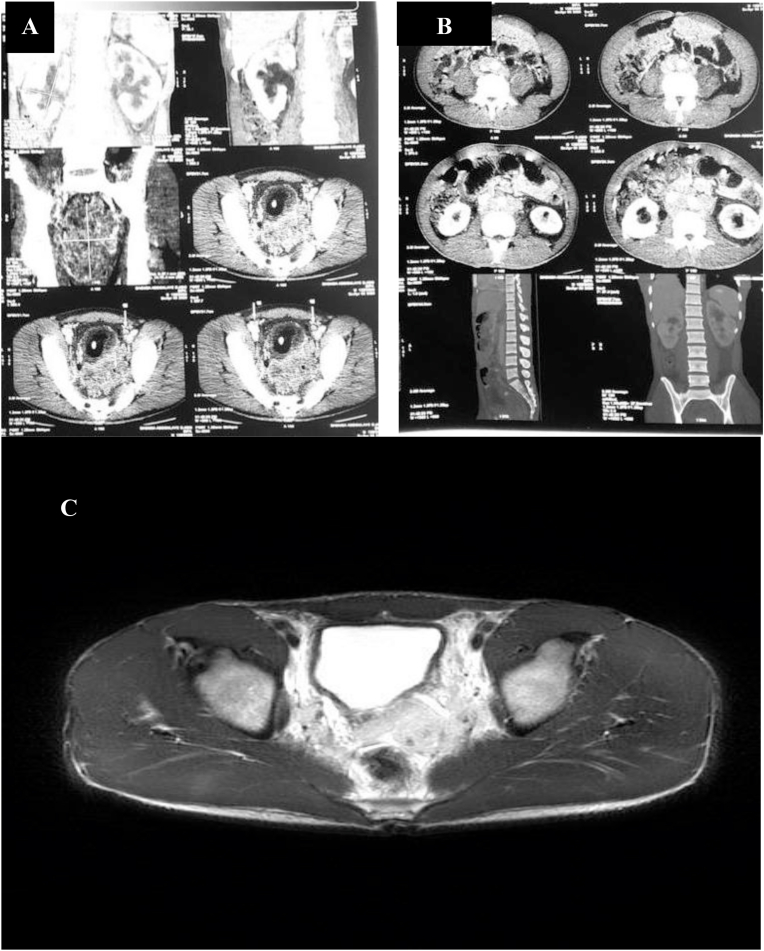
**(A)** Computed tomography (CT) scan of the abdomen and pelvis revealing a prostate tumor with bilateral ureterohydronephrosis and pelvic lymph node metastases **(B)** lumbo-aortic lymph node metastases and bone metastases **(C)** Pelvic Magnetic resonance imaging (MRI) showing a prostate tumor measuring 102× 82.6 × 64.4 mm with capsular crossing and invasion of the posterior bladder wall.

**Fig. 3:**
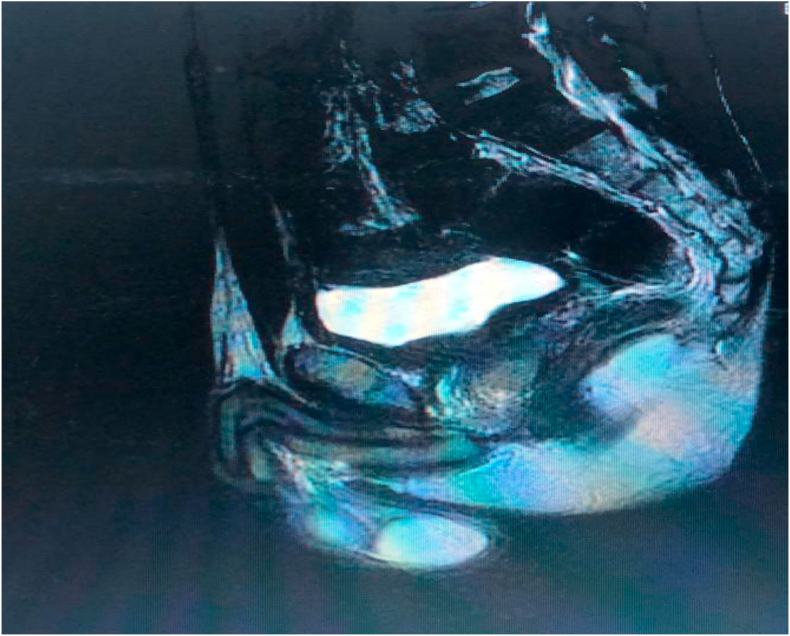
Pelvic Magnetic resonance imaging (MRI) showing a tumor melting and adenopathies disappearance.

A 26-year-old man, with no past medical and surgical history, consulted for an obstructive lower urinary tract involving urinary frequency, dysuria and urination imperiousness lasting for about six months. These symptoms were accompanied by anorexia, asthenia and a weight loss (7 kg in six months). The physical examination noticed a poor general condition with an ECOG score of 3. Rectal touch showed an uneven swollen and enlarged prostate gland of stony-hard consistency and nodular surface. Prostate-specific antigen (total PSA) level was 0.35 ng/ml and creatininemia was normal. Ultrasound of the urinary tract showed a heterogeneous prostate, enlarged, and whose weight was estimated at 123 g with stage II bilateral ureterohydronephrosis. A prostate cancer was suspected, given the characteristics of clinical and ultrasound of the prostate gland. A prostatic *trans*-rectal biopsy was performed and histology showed sheets of multinucleated cells in addition to small round cells (Fig. 1A). The round cells demonstrated immunohistochemical positivity for muscle-specific markers, such as myogenin, thus confirming the diagnosis of embryonal rhabdomyosarcoma (Fig. 1B). A computed tomography (CT) scan of the abdomen and pelvis revealed a prostate tumor with bilateral ureterohydronephrosis, pelvic lymph node metastases (Fig. 2A) and lumbo-aortic lymph node metastases and bone metastases (Fig. 2B). Pelvic Magnetic resonance imaging (MRI) scan showed a prostate tumor measuring 102× 82.6 × 64.4 mm with capsular crossing and invasion of the posterior bladder wall (Fig. 2C). The diagnosis was metastatic embryonal rhabdomyosarcoma of the prostate.Concurrent radio-chemotherapy was decided by the multidisciplinary consultation meeting. The patient had 9 chemotherapy treatments on doxorubicin (40mg), vincristine (1.5mg) and endoxan (800mg) associated with conformational radiotherapy on the prostate (70 Gy in 35 fractions). After treatment the urinary symptoms disappeared and the control pelvic MRI had shown a tumor melting and adenopathies disappearance (Fig. 3).After a one-year setback the patient has a good quality of life and no clinical and radiological recurrence has been detected at control imaging.

## Discussion

Prostate sarcoma is rare in adults and accounting for less than 5% of all prostate malignancies. . Rhabdomyosarcoma occurs mainly in children during the first decade with a peak between birth and 6 years of age. It represents the most common histological type of prostate sarcoma (30–40%). The embryonal subtype is the most common and is of better prognosis than botryoid, alveolar or polymorphic subtypes.^1^

The natural history of the disease is characterized by rapid progression.^2^ Typically there is extensive periurethral, perivesical, and perirectal invasion, with urethral obstruction, displacement of the bladder, or compression of the rectum. The presenting symptoms invariably are related to bladder outlet obstruction, with frequency, hesitancy, and dysuria the predominant symptoms, and with hematuria being uncommon. These typically progress rapidly to acute urinary retention. Invasion of the bladder is common, which may make proof of origin from the prostate impossible in more advanced tumors. Rectal examination reveals an enlarged prostate with an irregular nodular hard surface as in our patient. The tumor often disseminates widely, mainly to the lungs, bone, liver, and serosal surfaces.Metastases being generally osteolastic as in our patient.^2^Contrary to most other sarcomas, regional lymph node metastases are frequent.^2^

In most cases, PSA levels are not informative, and normality can cause a wrong diagnosis like in the case our patient. Pelvic abdomino CT scan with contrast product injection allows the diagnosis of the tumor developed at the expense of the prostate, the scannographic aspect is not sufficient to identify the sarcomatious nature, even if it can guide the diagnosis according to the context (age, tumor size). Magnetic Resonance Imaging is useful for studying relationships with adjacent organs, in the event of a surgical gesture. Computerised tomography reveals a large soft tissue mass with areas of necrosis replacing whole of prostate. Typically there is extensive invasion of bladder base and perirectal tissue planes MRI (magnetic resonance imaging) shows infiltration of surrounding bladder base, Dononvillier's fascia and rectal wall and levator anni muscle as is seen in our patient. The radiological investigations are helpful in characterizing the primary tumor and in detecting spread to the regional nodes.Immunohistochemistry is used to establish the diagnosis, which is based on immunopositivity for desmin and skeletal muscle markers.^2^In our patient, histological examination coupled with immunohistochemistry allowed the diagnosis of embryonal rhabdomyosarcoma.

The choice of treatment depends on the stage of the disease.^3^ In localised disease, radical surgery should be performed, but in locally advanced disease, surgery can cause severe impairment of function and is of questionable therapeutic value.^3^ As an alternative to surgery, radiotherapy combined with chemotherapy could be useful to achieve local control with functional preservation of organs and good quality of life. For metastatic stages, the impact and optimal timing of local therapies is unknown.^4^ A recent study showed that for metastatic rhabdomyosarcoma, radiotherapy was the only significant factor in improving progression-free survival and overall survival.^4^ Ashlock et al. reported improved survival and quality of life outcomes in children treated with multimodality therapy.^5^However, in adults the prognosis remains poor. The 70 Gy dose delivered to our patient allowed local control of the disease without toxicity. Similarly, the chemotherapy regimen adopted (doxorubicin, vincristine, endoxan) was initially useful. In our experience, concomitant chemo-radiotherapy provided an effective approach in an adult patient with metastatic rhabdomyosarcoma of the prostate. No clinical and radiological recurrence has been detected at control imaging. Clinically the patient had a good general state, the urinary symptoms disappeared.

## Conclusion

Prostate rahdomyosarcomas rare mesenchymal malignancies in adults with a poor prognosis. The stage influences the outcome. In metastatic forms the poor prognosis should change with improved and standardised radiochemotherapy regimens.

## Consent

Informed consent was provided by the patient.

## Funding sources

This research did not receive any specific grant from funding agencies in the public, commercial, or not-for-profit sectors.

## Declaration of competing interest

The authors have no conflicts of interest to declare.
